# Neuropsychiatric symptoms across levels of cognition in Alzheimer's disease in Down Syndrome and late‐life sporadic Alzheimer's disease

**DOI:** 10.1002/alz70857_101619

**Published:** 2025-12-25

**Authors:** Christina M Magana‐Ramirez, Daniel L Gillen, Christy L. Hom, Lidio Jaimes, David L Sultzer, Srisai Saavarni Chilukuri, Noah McMahon, Casie Lin, Ryan Kuhlman, Tiffany M Hu, Elizabeth Head, Ira T. Lott

**Affiliations:** ^1^ University of California, Irvine, Irvine, CA, USA; ^2^ The UC Irvine Institute for Memory Impairments and Neurological Disorders, Irvine, CA, USA; ^3^ The UC Irvine Institute for Alzheimer's Disease Research Center, Irvine, CA, USA; ^4^ University of Georgia, Athens, GA, USA; ^5^ School of Medicine, University of California, Irvine, Irvine, CA, USA; ^6^ University of California, Riverside, Riverside, CA, USA; ^7^ University of Washington, Seattle, WA, USA; ^8^ University of Florida, Gainesville, FL, USA; ^9^ University of California, San Diego, San Diego, CA, USA; ^10^ Alzheimer Biomarker Consortium‐Down Syndrome, Irvine, CA, USA

## Abstract

**Background:**

Alzheimer's disease (AD) affects older adults and individuals with Down syndrome (DS). While neuropsychiatric symptoms (NPS) often precede cognitive decline in late‐onset sporadic AD (LOAD), NPS in Down syndrome AD (DSAD) are less understood. We compared NPS between DSAD and LOAD to enhance understanding of AD pathophysiology and improve clinical care.

**Method:**

We used data from National Alzheimer's Coordinating Center (NACC) Uniform Dataset and the Alzheimer's Biomarker Consortium‐Down Syndrome (ABC‐DS), both of which administer the Neuropsychiatric Inventory (NPI) to measure neuropsychiatric symptoms (NPS). We calculated a composite NPI score (0‐36). We categorized participants as cognitively normal/stable (CN/CS), mild cognitive impairment (MCI), or demented (DEM). We used linear regression and generalized estimating equations to analyze the relationship between cognitive status and NPI scores, and NPI score trajectories over time, respectively, comparing DSAD and LOAD while adjusting for confounders.

**Result:**

We observed a homogenous relationship between NPI total score and cognitive status across the LOAD and DSAD syndromes through a multivariate Wald test at *p* = 0.282. We estimated a mean difference in NPI total score across LOAD and DSAD groups was ‐0.208 (95% CI: [‐0.535, 0.119]; *p* = 0.212); ‐0.034 (95% CI: ‐1.049, 0.982]; *p* = 0.948); and 0.731 (95% CI: [‐0.395, 1.857]; *p* = 0.203) for CN/CS, MCI, and DEM cognitive status, respectively. In our longitudinal analysis, a multivariate Wald test (*p* = 0.516) indicated the association between cognitive status and the rate of change in NPI total scores was overall homogeneous. The estimated mean difference in the NPI rate of change comparing LOAD to DSAD groups was: 0.286 (95% CI: [0.12, 0.453]; *p* = 0.001); 0.352 (95% CI: [0.021, 0.682]; *p* = 0.037); and ‐0.019 (95% CI: [‐0.507, 0.469 ]; *p* = 0.939) for CN/CS, MCI, DEM disease stages, respectively.

**Conclusion:**

Our findings suggest similar relationships between cognitive status and NPS severity across syndromes, suggesting that the shared amyloid pathology may be contributing across the two groups. These results also highlight the importance of NPS in early diagnosis, treatment, and caregiving. Further research is needed to validate and expand upon these findings.

